# The Skin in Winter

**Published:** 1908-02-01

**Authors:** 


### Dermatology.

### THE SKIN IN WINTER.

To many persons with very sensitive skins the winter season proves to be a time of real hardship. Sharp frosts or cutting winds have an unpleasant way of finding out the weak points in the cutaneous system, and unless special attention is paid to the hygiene of the skin a good deal of unnecessary discomfort, or even actual suffering, must needs be endured. The effect of cold upon the normal skin varies according to its intensity, the duration of its application, and the resisting power of the individual. As an immediate result of exposure to cold the minute papillary vessels in the cutis are constricted, producing a more or less intense anæmia, which, in its turn, gives place to some degree of reactionary erythema. This constitutes the "fresh" glow seen upon the face of a healthy person after active exercise on a cold day, best marked, naturally, in the young. If the peripheral circulation be weak owing to deficient cardiac action or to lack of arterial tone, this characteristic reaction either is very poor or may be altogether absent. One of the most obvious forms of dermatitis resulting from exposure to cold is

### The Common Chilblain, which is not often regarded seriously unless ulceration be a marked feature. It would seem as though chilblains more often make their first appearance at the onset of cold and damp weather than during actual frost. The hands, face and feet are the parts most usually affected in order of frequency. When they occur in an ulcerated condition at or about the insertion of the tendo Achillis the old name of "kibes" is still applied to them in some quarters. Erythema pernio is, perhaps, the chief bane of sanatorium life in the winter, rendering this form of treatment far more unpleasant than it would otherwise be. However, much can be done in the way of prevention. The extremities should be kept as warm and dry as possible; damp boots in particular are bad. The hands should be washed in warm water and thoroughly dried, but not at a fire. A little tincture of iodine or simple collodion may be applied at night. If ulceration should occur it must be treated antiseptically with lotions or fomentations, followed by some soothing ointment. It is often most advantageous to give five or ten grains of calcium lactate three times a day. A chronically congested state of the hands is sometimes seen, even in those who never suffer from chilblains. This condition, known as erythema perstans, is more noticeable in cold weather, but it is more or less persistent all the year round. The circulatory system in general should receive special attention in such cases, and small doses of strophanthus may be found necessary. Frost-bite may be regarded as an exaggerated chilblain.

A certain form of eczema may be distinctly attributable to extreme cold, and a papular or squamous dermatitis, to which the name "dermatitis hiemalis" has been applied by Corlett, may attack the face, neck, or, indeed, any part of the body. Those patients who are liable to an attack of ordinary eczema find sharp, biting winds very trying, and, similarly, the individual with chronic xerodermia is particularly apt to develop an eczema upon the exposed parts of the skin. The greatest care should be taken by such in relation to the daily hygiene of the skin. Superfatted soaps containing cold cream and oatmeal will be found better than the ordinary kind, but in many cases the use of oatmeal itself in the form of powder is preferable even to a special soap. Careful and thorough drying must be insisted upon, after which a little protective powder may be sprinkled over the surface. Ladies should wear veils when out of doors, and gentlemen should employ a dusting-powder after shaving.

### Chapped Hands.

One of the minor ills to which the skin is heir is the fissuring and cracking of the integument over the knuckles, around the nail-beds, and upon the sensitive finger-tips ocurring in cold weather. Sometimes these abrasions seem to make their appearance in spite of every care exercised in the toilet, and they may be so painful as to interfere seriously with the use of the fingers in certain fine movements—*e.g.* in violin-playing, etc. The judicious nightly use of glycerine and rose-water, or, better still, glycerine jelly, well rubbed in, will cure and certainly prevent most cases of chapped hands, but sometimes a little zinc and lanoline ointment may prove more soothing. The chronic fissures met with occasionally around the margins of the lips may be lightly touched with a weak solution of silver nitrate, after which some boric-acid ointment may be applied. Cold or very hot water should not be used for washing the face or hands, and oatmeal powder may be substituted for soap. It need hardly be stated that warm gloves are essential in the treatment and prevention of skin affections of the hands in winter. Only in very severe or neglected cases do "chaps" upon the hands become secondarily infected.

A special variety of pruritus, peculiar to cold weather, was described by Duhring, and afterwards by Hutchinson, known as pruritus hiemalis, or winter itch. It usually comes on at night when the patient is undressing to go to bed, when quite a paroxysm of itching commences. This may last for some time, and it is apt to recur every night, but the affection generally disappears completely with the first warm days of spring. In character this type is allied to the so-called bath-pruritus, and it is most probably due to the direct effect of cold air upon the pilo-sebaceous mechanism. Weak carbolic acid or alkaline lotions, diluted with warm water and freely dabbed upon the pruriginous areas, are indicated in this affection, which, if of less practical importance owing to its comparative rarity than the above-mentioned conditions, is nevertheless one which causes considerable discomfort to the sufferer therefrom. Cutaneous therapy in winter may thus be summed up—adequate protection, the regular use of emollients, and a sufficiency of bodily exercise in order to ensure a good peripheral circulation.

